# Multicenter Propensity Score-Based Study of Laparoscopic Repeat Liver Resection for Hepatocellular Carcinoma: A Subgroup Analysis of Cases with Tumors Far from Major Vessels

**DOI:** 10.3390/cancers13133187

**Published:** 2021-06-25

**Authors:** Arimasa Miyama, Zenichi Morise, Luca Aldrighetti, Giulio Belli, Francesca Ratti, Tan-To Cheung, Chung-Mau Lo, Shogo Tanaka, Shoji Kubo, Yukiyasu Okamura, Katsuhiko Uesaka, Kazuteru Monden, Hiroshi Sadamori, Kazuki Hashida, Kazuyuki Kawamoto, Naoto Gotohda, KuoHsin Chen, Akishige Kanazawa, Yutaka Takeda, Yoshiaki Ohmura, Masaki Ueno, Toshiro Ogura, Kyung-Suk Suh, Yutaro Kato, Atsushi Sugioka, Andrea Belli, Hiroyuki Nitta, Masafumi Yasunaga, Daniel Cherqui, Nasser Abdul Halim, Alexis Laurent, Hironori Kaneko, Yuichiro Otsuka, Ki-Hun Kim, Hwui-Dong Cho, Charles Chung-Wei Lin, Yusuke Ome, Yasuji Seyama, Roberto I. Troisi, Giammauro Berardi, Fernando Rotellar, Gregory C. Wilson, David A. Geller, Olivier Soubrane, Tomoaki Yoh, Takashi Kaizu, Yusuke Kumamoto, Ho-Seong Han, Ela Ekmekcigil, Ibrahim Dagher, David Fuks, Brice Gayet, Joseph F. Buell, Ruben Ciria, Javier Briceno, Nicholas O’Rourke, Joel Lewin, Bjorn Edwin, Masahiro Shinoda, Yuta Abe, Mohammed Abu Hilal, Mohammad Alzoubi, Minoru Tanabe, Go Wakabayashi

**Affiliations:** 1 Department of Surgery, Okazaki Medical Center, Fujita Health University School of Medicine, Okazaki 444-0827, Japan; arimasamiyama@gmail.com; 2 Hepatobiliary Division in Department of Surgery, San Raffaele Hospital, 20132 Milano, Italy; aldrighetti.luca@hsr.it (L.A.); ratti.francesca@hsr.it (F.R.); 3 Department of General and HPB Surgery, Loreto Nuovo Hospital, 80121 Naples, Italy; chirurgia.loretonuovo@tin.it; 4 Division of HBP and Liver Transplant, University of Hong Kong Queen Mary Hospital, Hong Kong 999077, China; tantocheung@hotmail.com (T.-T.C.); chungmlo@hku.hk (C.-M.L.); 5 Department of Hepato-Biliary-Pancreatic Surgery, Graduate School of Medicine, Osaka City University, Osaka 545-8586, Japan; m8827074@msic.med.osaka-cu.ac.jp (S.T.); m7696493@msic.med.osaka-cu.ac.jp (S.K.); 6 Division of Hepato-Biliary-Pancreatic Surgery, Shizuoka Cancer Center Hospital, Shizuoka 411-8777, Japan; yu.okamura@scchr.jp (Y.O.); k.uesaka@scchr.jp (K.U.); 7 Departments of Surgery, Fukuyama City Hospital, Fukuyama 721-8511, Japan; monden0319@yahoo.co.jp (K.M.); sadamorih@yahoo.co.jp (H.S.); 8 Department of Surgery, Kurashiki Central Hospital, Kurashiki 710-8602, Japan; kh14813@kchnet.or.jp (K.H.); kk7159@kchnet.or.jp (K.K.); 9 Division of Hepatobiliary and Pancreatic Surgery, National Cancer Center Hospital East, Kashiwa 277-8577, Japan; ngotohda@east.ncc.go.jp; 10 Division of General Surgery, Department of Surgery, Far-Eastern Memorial Hospital, New Taipei City 220, Taiwan; chen.kuohsin@gmail.com; 11 Department of Electrical Engineering, Yuan Ze University, Chung-Li 32003, Taiwan; 12 Department of Hepato-Biliary-Pancreatic Surgery, Osaka City General Hospital, Osaka 534-0021, Japan; kanazawaaki@mac.com; 13 Department of Surgery, Kansai Rosai Hospital, Amagasaki 660-8511, Japan; takeda-yutaka@kansaih.johas.go.jp (Y.T.); ohmura-yoshiaki@kansaih.johas.go.jp (Y.O.); 14 Second Department of Surgery, Wakayama Medical University, Wakayama 641-5810, Japan; ma@wakayama-med.ac.jp; 15 Department of Hepatobiliary and Pancreatic Surgery, Graduate School of Medicine, Tokyo Medical and Dental University, Tokyo 113-8519, Japan; ogumsrg@tmd.ac.jp (T.O.); tana.msrg@tmd.ac.jp (M.T.); 16 Department of Hepatobiliary and Pancreatic Surgery, Seoul National University Hospital, Seoul 03080, Korea; kssuh2000@gmail.com; 17 Department of Gastrointestinal Surgery, School of Medicine, Fujita Health University, Toyoake 470-1192, Japan; y-kato@fujita-hu.ac.jp (Y.K.); sugioka@fujita-hu.ac.jp (A.S.); 18 Department of Abdominal Surgical Oncology, Fondazione G.Pascale-IRCCS, National Cancer Institute of Naples, 80131 Napoli, Italy; a.belli@istitutotumori.na.it; 19 Department of Surgery, Iwate Medical University, Iwate 028-3695, Japan; hnitta@iwate-med.ac.jp; 20 Department of Surgery, School of Medicine, Kurume University, Kurume 830-0011, Japan; yasunaga@med.kurume-u.ac.jp; 21 Department of Hepato-Biliary Surgery and Transplantation, Hepatobiliary Centre, Paul Brousse Hospital, Villejuif 94800, France; daniel.cherqui@aphp.fr (D.C.); shac.x15@gmail.com (N.A.H.); 22 Department of Digestive, Hepatobiliary, Pancreatic Surgery and Liver Transplantation, Hôpital Henri Mondor, Assistance Publique-Hôpitaux de Paris, 94010 Créteil, France; alexis.laurent@aphp.fr; 23 Division of General and Gastroenterological Surgery, Department of Surgery, Toho University Faculty of Medicine, Tokyo 143-8541, Japan; hironori@med.toho-u.ac.jp (H.K.); yotsuka@med.toho-u.ac.jp (Y.O.); 24 Division of Hepatobiliary Surgery and Liver Transplantation, Department of Surgery, Ulsan University and Asan Medical Center, Seoul 05505, Korea; khkim620@amc.seoul.kr (K.-H.K.); hwuidongcho@gmail.com (H.-D.C.); 25 Department of Surgery and Surgical Oncology, Koo Foundation Sun Yat-Sen Cancer Center, Taipei 11259, Taiwan; charleslin@ircadtaiwan.com.tw; 26 IRCAD-AITS, Changhua County 505, Taiwan; 27 Department of Surgery, Tokyo Metropolitan Cancer and Infectious Diseases Center Komagome Hospital, Tokyo 113-8677, Japan; yusuke_omen@yahoo.co.jp (Y.O.); seyamaysur-tky@umin.ac.jp (Y.S.); 28 Department of Clinical Medicine and Surgery, University of Naples Federico II, 80138 Napoli, Italy; roberto.troisi@unina.it; 29 General Hepato-Biliary and Liver Transplantation Surgery, Ghent University Hospital Medical School, 9000 Gent, Belgium; gberardi1@gmail.com; 30 HPB and Liver Transplant Unit, Clinica Universitaria de Navarra, 31008 Pamplona, Spain; frotellar@gmail.com; 31 Department of Surgery, University of Pittsburgh, Pittsburgh, PA 15260, USA; wilsongc@upmc.edu (G.C.W.); gellerda@upmc.edu (D.A.G.); 32 Department of HPB Surgery and Liver Transplant, Beaujon Hospital, 92110 Clichy, France; olivier.soubrane@gmail.com (O.S.); tomoakiyoh@gmail.com (T.Y.); 33 Department of Surgery, School of Medicine, Kitasato University, Sagamihara 252-0375, Japan; t-kaizu@kitasato-u.ac.jp (T.K.); kumamoto@kitasato-u.ac.jp (Y.K.); 34 Department of Surgery, College of Medicine, Seoul National University, Bundang Hospital, Gyeonggi-do, Seongnam 13620, Korea; hanhs@snubh.org (H.-S.H.); eekmekcigil@gmail.com (E.E.); 35 Department of Minimally Invasive Digestive Surgery, Antoine Béclère Hospital, 92140 Clamart, France; ibrahim.dagher@aphp.fr; 36 Department of Digestive Diseases, Institute Mutualiste Montsouris, University of Paris Descartes, 75014 Paris, France; davidfuks80@gmail.com (D.F.); Brice.Gayet@imm.fr (B.G.); 37 Department of Surgery, Tulane Transplant Abdominal Institute, Tulane University, New Orleans, LA 70118, USA; Joseph.Buell@hcahealthcare.com; 38 Unit of Hepatobiliary Surgery and Liver Transplantation, University Hospital Reina Sofia, 14004 Córdoba, Spain; rubenciria@gmail.com (R.C.); javibriceno@hotmail.com (J.B.); 39 Department of General Surgery and HPB Surgery, Royal Brisbane Hospital, The University of Queensland, Herston, Brisbane, QLD 4029, Australia; orourke.nick@gmail.com (N.O.); joel.lewin@uqconnect.edu.au (J.L.); 40 Department of Hepatopancreatobiliary Surgery, Oslo University Hospital-Rikshospitalet, 0372 Oslo, Norway; bjoedw@ous-hf.no; 41 Department of Surgery, School of Medicine, Keio University, Tokyo 160-8582, Japan; masa02114@yahoo.co.jp (M.S.); abey3666@gmail.com (Y.A.); 42 Department of Surgery, Istituto Ospedaliero–Fondazione Poliambulanza, 25124 Brescia, Italy; abuhilal9@gmail.com; 43 Department of Surgery, University Hospital of Southampton NHS Foundation Trust, Southampton SO16 6YD, UK; mhm0001900@yahoo.com; 44 Department of General Surgery, Jordan University Hospital, The University of Jordan, Amman 11942, Jordan; 45 Department of Surgery, Ageo Central General Hospital, Ageo 362-8588, Japan; go324@mac.com

**Keywords:** laparoscopic liver resection, repeat surgery, repeat liver resection, hepatocellular carcinoma, morbidity, short-term outcome, long-term outcome

## Abstract

**Simple Summary:**

Less morbidity is considered among the advantages of laparoscopic liver resection for HCC patients. However, our previous international, multi-institutional study of laparoscopic repeat liver resection (LRLR) failed to prove it. We hypothesize that these results may be since the study included complex cases performed during the procedure’s developing stage. To examine it, subgroup analysis based on propensity score were performed, defining the proximity of the tumors to major vessels as the complexity. A propensity score matching earned 115 each patient of LRLR and open repeat liver resection (ORLR) without the proximity to major vessels, and the outcomes were compared. With comparable operation time and long-term outcome, less blood loss and less morbidity were shown in LRLR group than ORLR. Even in its worldwide developing stage, LRLR for HCC patients could be beneficial in blood loss and morbidity for the patients with less complexity in surgery.

**Abstract:**

Less morbidity is considered among the advantages of laparoscopic liver resection (LLR) for HCC patients. However, our previous international, multi-institutional, propensity score-based study of emerging laparoscopic repeat liver resection (LRLR) failed to prove this advantage. We hypothesize that these results may be since the study included complex LRLR cases performed during the procedure’s developing stage. To examine it, subgroup analysis based on propensity score were performed, defining the proximity of the tumors to major vessels as the indicator of complex cases. Among 1582 LRLR cases from 42 international high-volume liver surgery centers, 620 cases without the proximity to major vessels (more than 1 cm far from both first–second branches of Glissonian pedicles and major hepatic veins) were selected for this subgroup analysis. A propensity score matching (PSM) analysis was performed based on their patient characteristics, preoperative liver function, tumor characteristics and surgical procedures. One hundred and fifteen of each patient groups of LRLR and open repeat liver resection (ORLR) were earned, and the outcomes were compared. Backgrounds were well-balanced between LRLR and ORLR groups after matching. With comparable operation time and long-term outcome, less blood loss (283.3±823.0 vs. 603.5±664.9 mL, *p* = 0.001) and less morbidity (8.7 vs. 18.3 %, *p* = 0.034) were shown in LRLR group than ORLR. Even in its worldwide developing stage, LRLR for HCC patients could be beneficial in blood loss and morbidity for the patients with less complexity in surgery.

## 1. Introduction

Hepatocellular carcinoma (HCC) is the most common primary liver malignancy [1,2] with the neoplastic background of chronic liver diseases (CLD) [3]. The CLD background can develop multifocal and metachronous oncogeneses and repeat liver resections (LR) are often applied [4]. The indications for laparoscopic LR (LLR) have been expanded with accumulated experiences and developed instruments [5,6,7,8]. Reports of laparoscopic repeat liver resections (LRLR) are also increasing.

We had conducted a retrospective, international propensity score-based study of repeat LR for HCC to clarify the indications and outcomes of LRLR for HCC [9]. The study, with 1582 repeat LRs for HCC at 42 global high-volume liver surgery centers, showed that LRLR was feasible for selected patients and not inferior to open procedures in both the short- and long-term outcomes. The study also showed the differences in experiences and indications of LRLR between the centers, which means this procedure is still in its developing stage worldwide.

Less morbidity has been considered among the advantages of LLR for HCC with CLD patients. However, our previous study of LRLR failed to prove this advantage. We hypothesize that these results may be since the study included complex LRLR cases performed during its international developing stage. To examine the hypothesis, subgroup analyses of cases with less complexity based on propensity score were performed, defining the proximity of the tumors to major vessels, which increases the operative technical difficulty in both open LR and LLR [10,11], as the indicator of complex cases.

## 2. Methods

### 2.1. Participating Centers and Total Patients

The study involved 42 high-volume liver surgery centers from around the world that provided data of patients who underwent repeat LR (RLR) for HCC between January 2007 and December 2017. Institutional Review Board (IRB) approval was obtained from the coordinating center, with a data transfer agreement and IRB approval having been provided by all centers.

The centers registered 1582 patients, 934 and 648 treated by open and laparoscopic RLR, respectively, including those who had undergone RLR previously. Each case was discussed under a multidisciplinary setting in each center, and each patient provided informed consent for the procedure.

This study confirmed to the ethical guidelines of Declaration of Helsinki and was retrospective in nature. Approval from the ethics committee of each institution was obtained (HM20-094 for primary investigator’s institution, FHU).

### 2.2. Data Collection, Division of Patients into Groups, and Comparative Analysis

The following data were obtained: patient characteristics (age, sex, body mass index (BMI), and preoperative performance status); indicators of preoperative liver function (presence of liver fibrosis, background liver inflammation, plasma total bilirubin level (mg/dL), plasma albumin level (g/dL), platelet count (/μL), and prothrombin time); preoperative ascites, encephalopathy, and varices (each expressed as the related Child-Pugh score); tumor characteristics (number of tumors, size (mm), and location (anterolateral vs. posterosuperior segments), proximity to major vessels (Hilar plate-second branches of Glissonian pedicle (≤1 cm or >1 cm) and first–second branches of Major hepatic veins (≤1 cm or >1 cm)); and surgical procedures (open or laparoscopic LR, type of resection (partial resection-segmentectomy, sectionectomy, resection of two or more sections), and the previous LR procedure (open vs. laparoscopic)).

Among 1582 RLR cases, 614 cases without the proximity to major vessels (more than 1 cm far from both first–second branches of Glissonian pedicles and major hepatic veins) were selected for this subgroup analysis. These patients were divided into LRLR (*n* = 328) and open RLR (ORLR, *n* = 286) patients.

#### 2.2.1. Propensity Score Matching (PSM) Analysis

For all 614 cases, propensity scores (PS) were calculated based on their patient characteristics, preoperative liver function, tumor characteristics and surgical procedures, which were listed previously, and matching was performed by the scores. Operative and short-term postoperative outcomes (intraoperative blood loss volume (mL), need for blood transfusion, operation time (minutes), 90-day morbidity (≥Clavien–Dindo Grade (CD) II), 90-day morbidity (≥CD IIIa), 90-day mortality, postoperative hospital stay (days), and overall survival after RLR) were assessed, comparing between LRLR and ORLR groups before and after matching.

#### 2.2.2. Inverse Probability Treatment Weighted Analysis

In addition to PSM, 90-day morbidity (≥CD II), 90-day morbidity (≥CD IIIa), and overall survival after RLR were also compared between original (before matching) LRLR and ORLR patients using inverse probability treatment weight (IPTW). For all 614 patients, probability of receiving LRLR was calculated with logistic regression analysis using the same covariates as used in PSM analysis. The IPTW for each patient was defined as 1/probability for LRLR or 1/(1-probability) for ORLR patients. Weighting the outcome from each patient out of total 614 patients using IPTW, 90-day morbidity (≥Clavien–Dindo Grade (CD) II), 90-day morbidity (≥CD IIIa), and overall survival after RLR were compared between the groups.

### 2.3. Statistical Analyses

Data are expressed as median or mean ± standard deviation or as the number of patients. Between-group differences in categorical variables were analyzed by Pearson’s chi-squared test or Fisher’s exact test with Yates correction, as appropriate. Between-group differences in continuous parametric variables were analyzed by unpaired Student’s *t*-test or ANOVA, and between-group differences in continuous non-parametric variables were analyzed by the Mann–Whitney or the Kruskal–Wallis test. The correlation between the parameters were analyzed using Pearson’s correlation coefficient. Statistical analyses were performed with SPSS Statistics 25 (IBM Corp., Armonk, NY, USA), and *p* < 0.05 was considered statistically significant.

Patients were assigned propensity scores based on the factors listed above except outcomes.

## 3. Results

The LRLR group generally had better outcomes than the ORLR with the differences of background factors before matching (Table 1 and Table 2).

One hundred and fifteen of each PS-matched patients’ groups of LRLR and ORLR were earned. After PSM, backgrounds were well-balanced between the LRLR and the ORLR groups, except for background liver condition (Table 3). 

**Figure 1:**
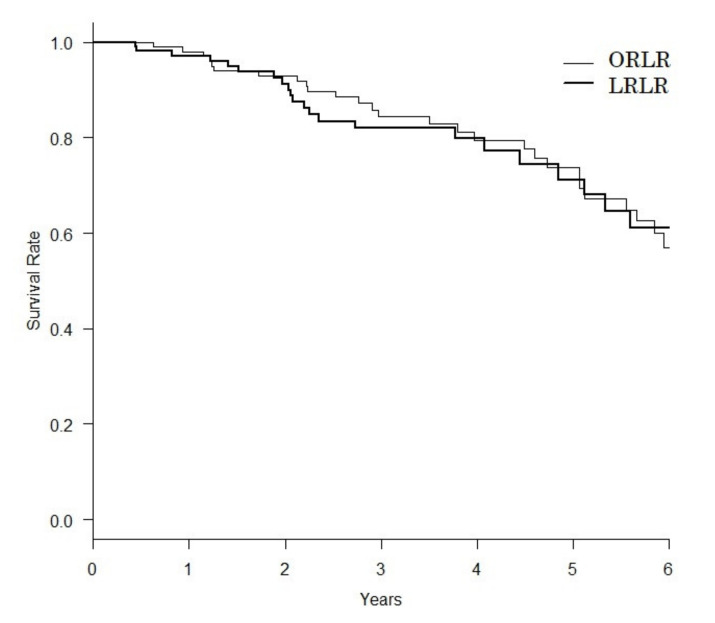
Overall survival curves after propensity score matching. Median overall survivals for open repeat liver resection (ORLR, thin line) and laparoscopic repeat liver resection (LRLR, thick line) were 7.51 and 7.14 years, respectively, and the difference of survival curves were not significant (*p* = 0.661).

The LRLR group matching had more patients with liver fibrosis or cirrhosis after. Less blood loss (283.3 ± 823.0 vs. 603.5 ± 664.9 mL, *p* = 0.001) and less morbidity (≥CD II, 8.7 vs. 18.3 %, *p* = 0.034; ≥CDIII, 4.3 vs. 12.2%, *p* = 0.031) were shown in the LRLR group than in the ORLR group. The operation times were comparable (260.6 ± 158.3 vs. 270.0 ± 129.6 mL, *p* = 0.622). The length of post-operative hospital stay was shorter, but not significant, in the LRLR group (10.2 ± 11.3 vs. 13.2 ± 12.1 mL, *p* = 0.058). As show in Table 4, the median overall survival for ORLR and LRLR were 7.51 and 7.14 years, respectively, and the difference of survival curves were not significant (*p* = 0.661, Figure 1).

The IPTW analyses for 614 original patients without matching showed less morbidity in LRLR patients (≥CD II, odd ratio = 0.540 (0.339–0.859), *p* = 0.009; ≥CDIII, 0.450 (0.255–0.797), *p* = 0.006) with comparable overall survival time (hazard ratio = 0.986 (0.721–1.349), *p* = 0.93).

## 4. Discussion

The present study showed a smaller amount of blood loss, less morbidity, a comparable operation time with comparable overall survival in LRLR patients with HCC more than 1 cm far from major vessels as compared to the ORLR counterpart. Although our previous study of 1582 RLRs for HCC [9] showed that LRLR was not inferior to ORLR and feasible for selected patients, the operation time was longer, and morbidity was similar in LRLR compared to the ORLR patients. The previous study also revealed notable differences in the indication of LRLR, even though all participants attended high-volume centers. The LRLR numbers and percentages among RLR had large differences between centers and, furthermore, the numbers and percentages were not correlated. This fact means that this procedure is applied depending on each centers’ experience and indication and the procedure is still in its developing stage worldwide, though it had been already a stable procedure in several institutions. The results of present study can be translated that LRLR for HCC patients could have merits in blood loss and morbidity without elongation of operation time for the patients with less complex surgery, even in its current developing stage. The length of hospital stay was also shorter (not significant) in the LRLR group of the present study, unlike the results from the previous study for the patients, including those with HCC near to major vessels.

The smaller amount of blood loss, lesser morbidity, and shorter length of hospital stays in LLR patients were shown in the first reported propensity score-based analysis of primary LLR and OLR for HCC [12]. However, a similar study for colorectal liver metastases failed to show decreased morbidity [13]. This has been thought to be due to the different liver backgrounds between these two most common diseases for LR. The minimal invasiveness of LLR can be more advantageous for CLD background in HCC patients [14]. Reports of LRLR are increasing [9,15,16,17,18,19,20], and investigators have noted its merits that LLR, with its magnified view, facilitates the meticulous dissection of adhesions strained by the pneumoperitoneum [16] and, furthermore, adhesiolysis can be avoided when the adhesion does not affect the operative procedure [15,18]. Other than our previous report, there are two propensity score matching analyses [21,22] and a meta-analysis of LRLR [23]. They reported smaller amount of blood loss and shorter hospital stay, but not less morbidity. However, they were the studies of HCC patients with CLD backgrounds. In our previous study for LRLR in HCC patients, less morbidity, which has been considered among the advantages of LLR for HCC with CLD patients [14], also failed to be proven. In order to examine the hypothesis that this may be due to the inclusion of complex cases performed during the procedure’s developing stage, subgroup analyses were performed in the present study, defining the proximity of the tumors to major vessels as the indicator of complex cases.

LRLR is generally applied to patients with poor liver function, under the consideration for the advantage of LLR with the preservation of the collateral vessels around liver and less damage to the adjacent structures. In the present study, the difference of background livers between LRLR and ORLR still existed after matching. However, it should have worked in favor of the ORLR group. Even under this condition, the LRLR group showed less morbidity after matching in addition to a smaller amount of blood loss. For the previous surgery after matching, there was no significant difference except for the tendency of LRLR patients having previous laparoscopic LR. Though it might reduce the amount of blood loss, the difference in blood loss was large between the LRLR and the ORLR groups, and our previous study [9] for total group patients has already shown reduced blood loss for LRLR patients. The tumor location and the extent of the resection in RLR were well-matched. However, we had the limitation of not having enough data of those in previous surgery. Shorter length of hospital stay should be one of the advantages of laparoscopic surgery. However, there was no significant difference in those between the LRLR and ORLR groups after matching in the present study, even with the tendency of shorter hospital stay in the LRLR group. There were large differences in hospital stay between centers, areas, and countries, possibly due to insurance systems and hospitalization practices. This might be the reason why the present study failed to show the significant difference in length of hospital stay.

In this subgroup analysis, advantages of LRLR over open, smaller amount of blood loss and less morbidity, were shown for the patients of less complex surgery without elongation of operation time nor deterioration of long-term outcome, though the procedure is still in its developing stage. Several institutions with advanced technique and many experiences in LRLR reported their promising results [15,16,17,18,19]. Although the procedure had been standardized with merits in those institutions, it is still the question to be investigated whether the procedure could become a common surgical procedure in many hospitals in the near future. The surprising speed of LLR development during quarter century from its start raises the expectations.

## 5. Conclusions

In this subgroup analysis, advantages of LRLR over open, smaller amount of blood loss and less morbidity, were shown for the patients of less complex surgery without elongation of operation time nor deterioration of long-term outcome, though the procedure is still in its developing stage.

## Figures and Tables

**Table 1:** Patient and tumor backgrounds before propensity score matching.

Backgrounds	ORLR (*n* = 287)	LRLR (*n* = 328)	*p* Value
**Age (years)**	67.3 ± 9.8	67.7 ± 10.8	0.665
**Sex ratio (Male:Female)**	222:61	252:74	0.734
**BMI (kg/m^2^)**	23.1 ± 3.6	23.9 ± 3.7	0.009
**Performance status**			
0	249	256	0.022
1	36	71	
2	1	3	
3	1	2	
**Number of tumors**	1.47 ± 1.13	1.22 ± 0.60	<0.001
**Tumor size (mm)**	17.5 ± 11.5	16.3 ± 12.0	0.222
**Tumor location**			
Anterolateral	178	247	0.001
Posterosuperior	109	85	
**Background liver pathology**			
NL	30	40	<0.001
CH	52	21	
LF	88	103	
LC	100	166	
**Total Billirubin (mg/dL)**	0.79 ± 0.44	0.82 ± 0.53	0.404
**Prothronbin time (C-P score)**			
1:>70% (<1.7)	269	313	0.983
2: 40–70% (1.7–2.3)	7	8	
3:<40 (>2.3)	3	3	
**Creatinine(mg/dL)**	0.88 ± 0.68	0.90 ± 0.60	0.606
**Albumin(g/L)**	4.05 ± 0.44	4.01 ± 0.47	0.255
**Platelet count (×10^3^/) μL**	165.5 ± 180.9	216.1 ± 350.7	0.028
**ICG R15**	14.6 ± 8.4	15.1 ± 10.1	0.537
**Ascites (C-P score)**			
1	282	314	0.011
2	5	19	
3	0	0	
**Encephalopathy (C-P score)**			
1	286	331	0.652
2	1	2	
3	0	0	
**Varices (C-P score)**			
1	256	288	0.033
2	11	29	
3	6	12	
**Extent of resection**			
Partial resection/segmentectomy	252	309	0.008
Sectionectomy	20	20	
≥2 sections	14	3	
**Previous surgery**			
Open Liver resection	241	120	<0.001
Laparoscopic liver resection	23	149	

ORLR, open repeat liver resection; LRLR, laparoscopic repeat liver resection; BMI, body mass index; NL, normal liver; CH, chronic hepatitis; LF, liver fibrosis; LC, liver cirrhosis; C-P score, Child-Pugh score; ICGR15, Indocyanine green retention rate at 15 min; Bold and plain letters [in the left end column] make the separation between heading and selective items.

**Table 2:** Short-term outcomes before propensity score matching.

Outcomes	ORLR (*n* = 286)	LRLR (*n* = 328)	*p* Value
**Intraoperative Blood Loss (mL)**	629.0 ± 882.3	246.6 ± 570.5	<0.001
**Blood transfusion**			
No	243	306	0.008
Yes	40	25	
**Operation time (min)**	261.1 ± 159.6	235.2 ± 144.7	0.036
**90-day morbidity ≥ CD II**			
No	243	296	0.059
Yes	42	32	
**90-day morbidity ≥ CD IIIa**			
No	260	316	0.008
Yes	25	12	
**90-day mortality**			
No	282	326	0.253
Yes	3	1	
**Postoperative hospital stay (days)**	13.5 ± 11.6	9.6 ± 8.7	<0.001

ORLR, open repeat liver resection; LRLR, laparoscopic repeat liver resection; Bold and plain letters [in the left end column] make the separation between heading and selective items.

**Table 3:** Patient and tumor backgrounds after propensity score matching.

Backgrounds	ORLR (*n* = 115)	LRLR (*n* = 115)	*p* Value
**Age (years old)**	67.5 ± 9.5	68.2 ± 10.3	0.565
**Sex ratio (Male: Female)**	94:21	91:24	0.618
**BMI (kg/m^2^)**	23.6 ± 3.5	23.6 ± 3.6	0.933
**Performance status**			
0	103	99	0.367
1	11	16	
2	1	0	
3	0	0	
**Number of tumors**	1.45 ± 1.05	1.28 ± 0.82	0.164
**Tumor size (mm)**	14.5 ± 9.0	14.9 ± 12.7	0.792
**Tumor location**			
Anterolateral	72	83	0.122
Posterosuperior	43	32	
**Liver fibrosis**			
1 NL	11	16	<0.001
2 CH	28	4	
3 LF	37	51	
4 LC	39	44	
**Total Billirubin (mg/dL)**	0.76 ± 0.36	0.82 ± 0.37	0.258
**Prothronbin time (C-P score)**			
1:>70% (<1.7)	109	109	0.842
2: 40–70% (1.7–2.3)	4	3	
3:<40 (>2.3)	2	3	
**Creatinine(mg/dL)**	0.85 ± 0.62	0.90 ± 0.82	0.667
**Albumin(g/L)**	4.09 ± 0.40	4.04 ± 0.46	0.382
**Platelet count (×10^3^/) μL**	145.0 ± 77.0	150.9 ± 157.1	0.715
**ICG R15**	15.6 ± 9.2	14.5 ± 10.3	0.378
**Ascites (C-P score)**			
1	115	113	0.155
2	0	2	
3	0	0	
**Encephalopathy (C-P score)**			
1	115	115	-
2	0	0	
3	0	0	
**Varices (C-P score)**			
1	109	109	1.000
2	5	5	
3	1	1	
**Extent of resection**			
Partial resection/segmentectomy	103	108	0.332
Sectionectomy	8	6	
≥2 sections	4	1	
**Previous surgery**			
Open Liver resection	101	90	0.053
Laparoscopic liver resection	14	25	

ORLR, open repeat liver resection; LRLR, laparoscopic repeat liver resection; BMI, body mass index; NL, normal liver; CH, chronic hepatitis; LF, liver fibrosis; LC, liver cirrhosis; C-P score, Child-Pugh score; ICGR15, Indocyanine green retention rate at 15 min; Bold and plain letters [in the left end column] make the separation between heading and selective items.

**Table 4:** Short-term outcomes after propensity score matching.

Outcomes	ORLR (*n* = 115)	LRLR (*n* = 115)	*p* Value
**Intraoperative Blood Loss (mL)**	603.5 ± 664.9	283.3 ± 823.0	0.001
**Blood transfusion**			
No	99	103	0.420
Yes	16	12	
**Operation time (min)**	270.0 ± 129.6	260.6 ± 158.3	0.623
**90-day morbidity ≥ CD II**			
No	94	105	0.034
Yes	21	10	
**90-day morbidity ≥ CD IIIa**			
No	101	110	0.031
Yes	14	5	
**90-day mortality**			
No	115	115	-
Yes	0	0	
**Postoperative hospital stay (days)**	13.2 ± 12.1	10.2 ± 11.3	0.058

ORLR, open repeat liver resection; LRLR, laparoscopic repeat liver resection; Bold and plain letters [in the left end column] make the separation between heading and selective items.

## Data Availability

The data presented in this study are available on request from the corresponding author.
